# A pilot voxel-based morphometry study of older adults after the PICMOR intervention program

**DOI:** 10.1186/s12877-021-02669-x

**Published:** 2022-01-19

**Authors:** Hikaru Sugimoto, Mihoko Otake-Matsuura

**Affiliations:** RIKEN Center for Advanced Intelligence Project, Nihonbashi 1-chome Mitsui Building, 15th floor, 1-4-1 Nihonbashi, Chuo-ku, Tokyo, 103-0027 Japan

**Keywords:** Cognitive intervention, Conversation, Executive function, Lateral prefrontal cortex, PICMOR, Voxel-based morphometry

## Abstract

**Background:**

Age-related decline in cognitive function, such as executive function, is associated with structural changes in the neural substrates, such as volume reductions in the lateral prefrontal cortex. To prevent or delay age-related changes in cognitive function, cognitive intervention methods that employ social activity, including conversations, have been proposed in some intervention studies. Interestingly, previous studies have consistently reported that verbal fluency ability can be trained by conversation-based interventions in healthy older adults. However, little is known about the neural substrates that underlie the beneficial effect of conversation-based interventions on cognitive function. In this pilot study, we aimed to provide candidate brain regions that are responsible for the enhancement of cognitive function, by analyzing structural magnetic resonance imaging (MRI) data that were additionally obtained from participants in our previous intervention study.

**Methods:**

A voxel-based morphometric analysis was applied to the structural MRI data. In the analysis, the regional brain volume was compared between the intervention group, who participated in a group conversation-based intervention program named Photo-Integrated Conversation Moderated by Robots (PICMOR), and the control group, who joined in a control program based on unstructured free conversations. Furthermore, regions whose volume was positively correlated with an increase in verbal fluency task scores throughout the intervention period were explored.

**Results:**

Results showed that the volume of several regions, including the superior frontal gyrus, parahippocampal gyrus/hippocampus, posterior middle temporal gyrus, and postcentral gyrus, was greater in the intervention group than in the control group. In contrast, no regions showed greater volume in the control group than in the intervention group. The region whose volume showed a positive correlation with the increased task scores was identified in the inferior parietal lobule.

**Conclusions:**

Although definitive conclusions cannot be drawn from this study due to a lack of MRI data from the pre-intervention period, it achieved the exploratory purpose by successfully identifying candidate brain regions that reflect the beneficial effect of conversation-based interventions on cognitive function, including the lateral prefrontal cortex, which plays an important role in executive functions.

**Trial registration:**

The trial was retrospectively registered on 7 May 2019 (UMIN Clinical Trials Registry number: UMIN000036667).

**Supplementary Information:**

The online version contains supplementary material available at 10.1186/s12877-021-02669-x.

## Background

Cognitive function, such as executive function, declines with advancing age [1–6]. Age-related decline in cognitive function is closely associated with structural changes in the neural substrates [7–15], such as decreased volume in the lateral prefrontal cortex [16–21]. To prevent or delay age-related changes in cognitive function, cognitive intervention methods that are based on social activity, such as conversations, have been proposed in some intervention studies [22]. Interestingly, there is a consistent behavioral finding from previous studies that verbal fluency task scores, reflecting executive control and verbal abilities [23], are enhanced by conversation-based interventions in healthy older adults [24, 25]. However, little is known about the neural substrates that underlie the beneficial effect of conversation-based interventions on cognitive function. This pilot study aimed to provide candidate brain regions associated with the enhancement of cognitive function, by collecting structural magnetic resonance imaging (MRI) data from participants in our previous intervention study [25] and comparing the regional brain volume between the intervention and control groups.

Photo-Integrated Conversation Moderated by Robots (PICMOR) is a group conversation-based intervention program that we have developed to train cognitive function in older adults [25]. The group conversation in PICMOR is characterized by strict time management and automatic turn-taking, which are enabled by a robot that acts as a chair. The conversation begins with a talking period, during which the person who is selected as a speaker of topics is prompted by the robot to talk about their daily life for a certain length of time, using photos they have prepared beforehand. The talking period is followed by a discussion period, during which the speaker of topics has to answer questions from the other members of the group. The group members, who are not assigned as the speaker, are required to listen attentively during the speaker’s talks and to ask questions during the discussion period. The robot has been designed to monitor the conversation in real-time and directly prompt and stop the participants’ utterances to balance the production of speech for each participant. The robot moderator enables us to force the participants to make a speech for a certain length of time and finish their talk when they have talked enough. We assumed that executive functions, such as flexibility, planning, working memory, and response inhibition, would be exercised by repeated training in group conversations in PICMOR because the participants are required to make a speech within a limited time, flexibly ask and answer questions, keep in mind and manipulate the information to ask questions, and refrain from interrupting other members of the group in the conversations. To examine the effect of PICMOR on cognitive function in healthy older adults, we previously conducted a randomized controlled trial (RCT) [25]. In the RCT, those who were assigned to the intervention group joined the PICMOR once a week for 12 weeks, whereas those who were assigned to the control group took part in a free conversation program, where unstructured conversations without robotic support were offered. Confirming our assumptions, a beneficial intervention effect was observed in the score of the phonemic verbal fluency task [26, 27], which is often used to measure executive control and verbal abilities [23]. Specifically, a significantly larger increase in the verbal fluency task scores throughout the intervention period was found in the intervention group than in the control group [25]. Based on this finding, we hypothesized that the behavioral effect would be reflected in differences in the brain network responsible for verbal fluency between the two groups that emerged after the intervention period.

To examine the possible difference in the functional network involved in verbal fluency, we conducted an additional experiment for the participants in our previous RCT, using resting-state functional MRI [28, 29]. Indeed, we found significant differences in the resting-state functional connectivity between the two groups, including a significantly higher resting-state functional connectivity between the left and right prefrontal cortex in the intervention group than in the control group [30]. Taken together with evidence from previous neuroimaging and neuropsychological studies that the prefrontal cortex plays an important role in executive functions [31–39] and that the volume of this region has been shown to increase in healthy older adults by an intervention program designed to train executive functions [40], we further hypothesized that this region would have a greater volume in the intervention group than in the control group.

Therefore, in the present study, we applied a voxel-based morphometric (VBM) analysis [41] to the structural MRI data which were obtained after the intervention [30], and examined a possible difference in the regional brain volume between the two groups. In the analysis, we explored the structural difference at the whole-brain level in order to examine the possibility that it could be observed in brain regions other than regions where group difference in resting-state functional connectivity was found, such as the lateral prefrontal cortex [30]. Although we cannot fully attribute any possible differences to the effects of PICMOR because of a lack of comparisons with MRI data from the pre-intervention period, we aimed to identify candidate brain regions that reflect the beneficial effect of conversation-based interventions on brain structures for future research.

## Methods

### Participants

We re-used the MRI data from our previous study [30]. The data were collected from 31 participants in the intervention group (15 women and 16 men; 20 people with education for 13 years and more; age, mean ± standard deviation [SD] = 72.84 ± 3.45 years) and 30 participants in the control group (17 women and 13 men; 17 people with education for 13 years and more; age, mean ± SD = 72.03 ± 2.72 years) who had taken part in our previous RCT [25]. As reported in our previous study [30], we found no significant difference in age, sex, and education level between the two groups. MRI data were not available for four RCT participants: one participant each in the intervention and control groups had claustrophobia, one participant in the control group had a heart pacemaker, and one participant in the control group declined to undergo MRI scanning [30].

### Intervention procedures

The intervention procedures are detailed in our previous studies [25, 30] and briefly described below. In the RCT, 72 community-living older adults were recruited from the Silver Human Resources Center and required to receive screening and baseline assessment by medical interviews, cognitive tests, and self-reported questionnaires. The following exclusion criteria were employed for subsequent intervention phases: dementia; neurological impairment; any disease or medication known to affect the central nervous system; scoring less than 24 in the Japanese version of the Mini-Mental State Examination (MMSE-J) [42]. After the baseline assessment, 65 participants, who were considered as eligible for the RCT, were randomly allocated to the intervention (*n* = 32) or control (*n* = 33) groups. After the intervention period, a post-assessment by cognitive tests was conducted for the participants. In addition, MRI data were acquired from 31 and 30 participants in the intervention and control group, respectively, as mentioned above.

During the intervention period, both the intervention and control groups took part in a group conversation once a week for 12 weeks. For the group conversation, 8 subgroups, each with 4 members, were formed in each experimental group (only 1 subgroup in the control group had 5 members). Participants in the intervention group joined the group conversation offered by PICMOR, which is characterized by robotic support (explained in detail below), whereas participants in the control group gathered to talk freely without robotic facilitation among the group members, as they would converse in their daily life.

In the group conversations offered by PICMOR, a robot played the role of a chairperson and encouraged one participant to make a speech within a limited time (1 min) about an event that they had experienced in daily life, using a photo they had prepared beforehand. The photo was displayed on the screen so that it was visible for all group members. The participant selected as a speaker of topics was asked to provide the other members of the group with a total of two events, using two photos (i.e., each speaker was given a total of 2 min to talk). During the 1-min talking period for each event, the other members had to listen carefully to ask questions in the following discussion period. After that, another member was assigned as the speaker of topics. This procedure was repeated until all members finished talking about their topics. The talking period was followed by a 2-min discussion period for each event, during which the speaker of topics was required to answer questions asked by the other members of the group (i.e., each speaker was given a total of 4 min in the discussion). This process was also repeated until all members finished discussing their experiences. In this period, the robot moderated the conversation in real-time by prompting and stopping the participants’ utterances to balance the amount of speech time for each participant. For instance, when the robot detected that one participant had spent less time talking than the other members of the group, it directly encouraged the participant to ask questions or give comments. The robot moderator enabled strict time management and automatic turn-taking based on the actual talking time of each person, which would be difficult for a human moderator.

As noted earlier, we hypothesized that executive functions, including flexibility, planning, working memory, and response inhibition, would be trained by the group conversations in PICMOR, compared to unstructured conversations in the control program, because the participants had to make a speech within a certain length of time (i.e., 1 min), flexibly ask and answer questions, keep in mind and manipulate the information to ask questions, and refrain from interrupting other members of the group. Consistent with this idea, a beneficial intervention effect was observed in the score of the phonemic verbal fluency task [25], in which participants were required to produce as many words as possible beginning with a specific letter within a limited time (1 min) [26, 27]. This behavioral result would be reasonable, given that successful performance of this task requires them to flexibly retrieve appropriate words, keep previously produced words in working memory, and suppress inappropriate words or task-irrelevant thoughts. Behavioral results of multiple cognitive and mental health tests, including the verbal fluency task [30], in the participants of this VBM study (i.e., 31 and 30 participants in the intervention and control group, respectively) are summarized in Additional file 1.

### MRI data acquisition and analysis

The procedure of MRI data acquisition is described in our previous study [30]. In short, a high-resolution T1-weighted image was scanned with the following parameters: repetition time = 6.41 ms, echo time = 3.00 ms, field of view = 24.0 cm × 24.0 cm, matrix size = 256 × 256, slice thickness/gap = 1.2/0 mm, and 170 sagittal slices. During the scanning, participants’ head motion was minimized by a belt and foam pads. MRI data were collected using a 3 T Philips Achieva MRI scanner in Advanced Imaging Center Yaesu Clinic, Tokyo. As previously reported in our study [30], we found no significant difference in the period from the last day of interventions to the day of MRI data collection between the intervention (mean ± SD = 9.67 ± 0.76 weeks) and control (mean ± SD = 9.68 ± 0.56 weeks) groups.

The structural MRI data were analyzed using Statistical Parametric Mapping 12 (SPM 12) (www.fil.ion.ucl.ac.uk/spm/) implemented in MATLAB. In the preprocessing of structural images, five steps were performed. First, the images were segmented into intracranial parts of gray matter (GM), white matter (WM), cerebrospinal fluid (CSF), and other non-brain structures, such as cranium and extracranial tissues, by the standard unified segmentation module [43]. Second, the registration process was implemented by the Diffeomorphic Anatomical Registration using Exponentiated Lie algebra (DARTEL) toolbox [44], which enabled us to achieve a higher accuracy of inter-subject registration. This process began with an import of the DARTEL form of individual segmented GM and WM images, and resulted in the generation of study-specific template images and flow fields data. Third, using this information, we spatially normalized individual segmented images into the Montreal Neurological Institute (MNI) space with a resolution of 1.5 × 1.5 × 1.5 mm^3^ voxels. Fourth, normalized images were modulated to ensure that relative GM and WM volumes were well preserved after spatial normalization. Finally, these images were smoothed with a Gaussian kernel of 8 mm full-width at half-maximum.

For statistical analyses of structural MRI data, a binarized mask image was made from the preprocessed images by the masking toolbox (www0.cs.ucl.ac.uk/staff/g.ridgway/masking/) [45]. Employing this mask image as an explicit mask, we conducted a two-sample *t*-test to identify group differences in regional brain volumes. In the unpaired *t*-test, the variances of data were assumed to be unequal between the two groups. Moreover, we performed a regression analysis using the difference in verbal fluency task scores between the pre- and post-intervention periods (i.e., post-minus pre-intervention) as the regressor. These models included participants’ age, sex, and education level (≥ 13 years or < 13 years) as nuisance covariates. Total intracranial volumes (GM + WM + CSF) were also entered into the models for global calculation, which enabled us to exclude global nuisance effects (see Additional file 2). In the present study, 1000-iteration Monte-Carlo simulations were employed to determine a sufficient voxel contiguity threshold [46], assuming a type I error voxel-level threshold of *p* = 0.001 and a cluster extent threshold of *p* < 0.05. Results indicated a cluster extent of 69 contiguous resampled voxels was sufficient to correct for multiple comparisons. Based on the simulations, clusters with at least 69 voxels were reported in this study. The anatomical sites of the reported regions in the present study were determined with reference to the SPM Anatomy Toolbox [47–49] and MRIcro (www.cabi.gatech.edu/mricro/mricro/).

## Results

**Fig. 1:**
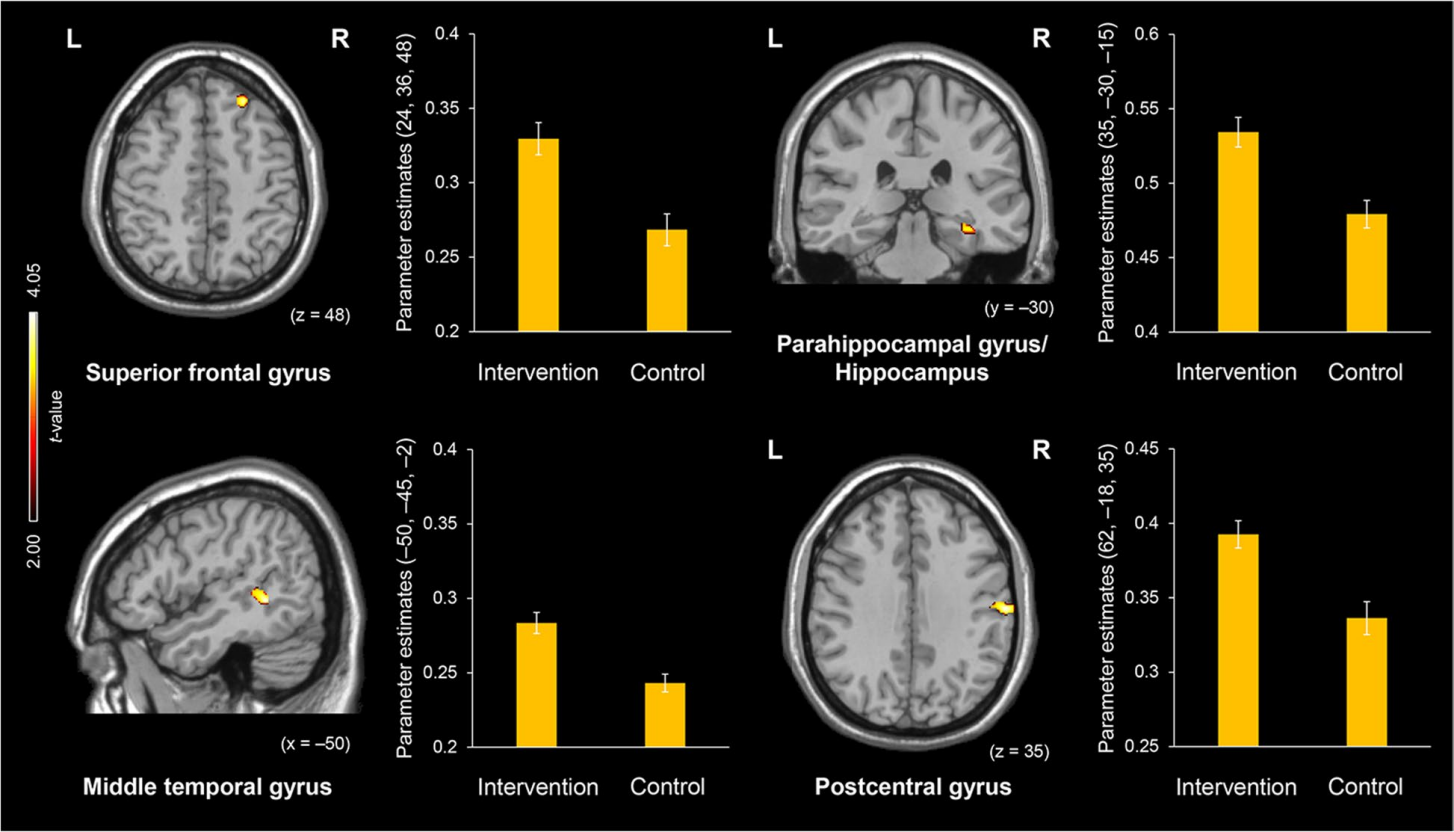
Regions showing significantly greater volume in the intervention group than in the control group. Notes: Error bars represent standard errors. Abbreviations: L, left; R, right

In the present study, the regional brain volume was compared between the intervention and control groups by a two-sample *t*-test. Consistent with our prediction, the right superior frontal gyrus, i.e., the lateral prefrontal cortex, showed a significantly greater volume in the intervention group than in the control group (Fig. 1). Other regions showing the same pattern were identified in the right parahippocampal gyrus/hippocampus, left posterior middle temporal gyrus, and right postcentral gyrus. In contrast, there were no regions showing significantly greater volume in the control group than in the intervention group. These findings are summarized in Table 1. Results from the regression analysis revealed that the volume of the right inferior parietal lobule was positively correlated with the increased verbal fluency task scores throughout the intervention period (x = 45, y = − 53, z = 51, BA 40, 78 voxels, Z value = 3.67).

**Table 1:** Regions showing a significant difference in volume between the intervention and control groups

Regions	L/R	BA	MNI coordinates			Voxel	Z value
			x	y	z		
***Intervention > Control***							
Superior frontal gyrus	R	9	24	36	48	85	3.65
Parahippocampal gyrus/Hippocampus	R		35	–30	–15	78	3.39
Middle temporal gyrus	L	21	−50	−45	−2	225	3.78
Postcentral gyrus	R	43	62	−18	35	140	3.75
***Control > Intervention***							
Not significant in any regions							

*Abbreviations*: *L* left, *R* right, *BA* Brodmann area, *MNI* Montreal Neurological Institute

## Discussion

This pilot study aimed to provide candidate brain regions that reflect the beneficial effect of conversation-based interventions on cognitive function in healthy older adults, by elucidating a possible difference in regional brain volumes between the intervention and control groups in our previous RCT [25]. The main finding was that the volume of the lateral prefrontal cortex in the intervention group was significantly greater than that in the control group. We also found that the volume of the medial temporal lobe, posterior middle temporal gyrus, and somatosensory cortex showed the same pattern. In contrast, no regions showed significantly greater volume in the control group than in the intervention group. Additionally, the volume of the inferior parietal lobule showed a positive correlation with the increased verbal fluency task scores throughout the intervention period. The present findings imply a possible intervention effect of PICMOR on brain structures. The findings are discussed in detail below.

Our main finding of a significantly greater volume in the lateral prefrontal cortex in the intervention group is consistent with evidence from a previous intervention study, which found that the volume of this region increased in healthy older adults who participated in a music training program designed to train executive functions [40]. Available evidence from neuropsychological studies of patients with brain lesions and neuroimaging studies of healthy adults supports the idea that the prefrontal cortex is involved in executive functions, such as flexibility, planning, working memory, and response inhibition [31–39]. Given that these executive functions would be exercised in the group conversations offered by PICMOR, in which participants have to make a speech within a limited time, flexibly ask and answer questions, intentionally store and manipulate the information to ask questions, and inhibit their interruption of other members [25, 30], it is reasonable that a significant group difference in volume was identified in this region. Taken together with our previous evidence that the resting-state functional connectivity between the left and right prefrontal cortex was significantly higher in the intervention group than in the control group [30], the present finding extends the previous finding by demonstrating that the group difference in the brain region responsible for executive functions emerged at the structural level. Significance of the current VBM finding of the lateral prefrontal cortex could be further supported by an additional analysis for the behavioral measure of multiple cognitive tests before and after the intervention period, in which significant difference in the verbal fluency scores between the two groups was found after the intervention, but not before the intervention (see Additional file 1) [30]. Given that the participants were randomly assigned to the intervention or control group before the intervention [25], and that there was no significant group difference in the verbal fluency scores before the intervention, it is unlikely that the present finding of the lateral prefrontal cortex reflects an intrinsic difference in the volume of this region between the two groups. From these reasons, it would be reasonable to suppose that the lateral prefrontal cortex is one of the candidate brain regions that are responsible for the enhancement of cognitive function by conversation-based interventions.

In this study, the volume of the inferior parietal lobule showed a positive correlation with the increase in the verbal fluency task scores throughout the intervention period. This finding is consistent with those of previous neuroimaging studies showing the contributions of the parietal region to switching during verbal fluency tasks [50–52]. Switching is a strategy for successful performance in verbal fluency tasks, which involves consecutively generating words not belonging to the same category [53]. This strategy requires effortful search processes, including cognitive flexibility [53], and has been associated with increased activation in the parietal lobe [50–52]. Taken together, the present finding that individuals with higher performance gains in the verbal fluency task had greater volume in the parietal region could reflect individual differences in the switching ability improved by interventions.

The volume of the medial temporal lobe has been shown to increase or be maintained in healthy older adults, who participated in other intervention programs designed to improve memory functions [40, 54, 55]. For example, one intervention study reported that healthy older adults, who joined in a memory training program, showed significantly greater increases in hippocampal volume than those who did not receive such training and patients with subjective memory impairments who received the same training [54]. In another study, both of younger and older adults, who participated in a spatial navigation training program, showed stable hippocampal volumes throughout the intervention period, whereas those who joined in a control program exhibited decreases in the volume [55]. Similar to previous intervention programs, the PICMOR was designed to exercise medial temporal lobe-dependent memory functions during the intervention period. In our previous RCT, participants in the intervention group were repeatedly trained by robotic support to talk about their daily life for a certain length of time, while those in the control group did not receive such training [25]. Talking about one’s own experiences undoubtedly requires the retrieval of autobiographical memories. Also, participants in the intervention group were trained to listen carefully to others and to ask them questions [25]. These aspects should involve the encoding of novel information from others and integration of this information. Given the difference in training demands between the two groups, it might be reasonable that a significant group difference was observed in the medial temporal lobe, as this region plays an important role in retrieval and encoding of episodic memories [56–59]. However, the present VBM finding of the medial temporal lobe could be limited by behavioral evidence that there was a significant group difference in the scores of the logical memory subtests (Logical memory I & II) from the Wechsler Memory Scale-Revised (WMS-R) [60] before the intervention period (see Additional file 1). This behavioral result suggests that the group difference in the volume of this region had already existed even before the intervention period. Further investigation is needed to examine whether the intervention effects could be reflected in the volume of the medial temporal lobe.

The left posterior middle temporal gyrus is included in a classical language-related region, termed Wernicke’s area [61]. Previous neuropsychological studies have consistently reported that patients with lesions in this region show impairment in language comprehension [62–64]. Although the precise functional contribution of the posterior middle temporal gyrus to the understanding of spoken language is still a matter of debate [65], one possible explanation by the Dual Stream Model of speech perception is that this region plays a pivotal role in language comprehension as a lexical interface, which matches the phonological structures of perceived words with the corresponding semantic structures [66–68]. As noted above, participants in the intervention group were trained to listen carefully to other members of the group and to ask them questions in the group conversations [25]. This aspect of training requires participants to comprehend the conceptual content of others’ utterances. From the perspective of the Dual Stream Model [66–68], the present VBM finding of the left posterior middle temporal gyrus could be interpreted as evidence of an enhanced or maintained ability of the intervention group to map phonological structures of utterances by the other members of the group onto their semantic structures during spoken language comprehension.

In this study, regions whose volume was different between the two groups were identified in several areas. However, almost all regions were not identical to the regions where the group difference in resting-state functional connectivity was observed [30]. This discrepancy could be derived from the difference in analytical approaches adopted in the current VBM study and the previous resting-state functional MRI study [30]. The previous study employed a seed-based approach [28, 29], which focuses on resting-state functional connectivity with specific brain areas as seed regions (in this case, the seeds were defined as spheres with a 5-mm radius around specific coordinates located in the left inferior and middle frontal gyri) [30] to characterize the functional network involved in verbal fluency, whereas this study adopted an unbiased approach by exploring group difference in brain volume at the whole-brain level to examine the possibility that the difference could be identified in brain regions other than the regions where group difference in resting-state functional connectivity was observed. Although comprehensive interpretations for the findings from both studies are limited due to this discrepancy, it is noteworthy that both studies supported the significance of the dorsolateral prefrontal cortex. Taken together with ample evidence regarding the contributions of the lateral prefrontal cortex to executive functions [31–39], this region could be modulated in both structural and functional levels by interventions designed to train executive functions.

There are two limitations in this study. One is a lack of comparable MRI data from the pre-intervention period. Although we found a significant group difference in regional brain volumes, we could not determine whether this difference reflects a relatively larger increase in the intervention group than in the control group or a smaller decrease in the intervention group than in the control group, throughout the intervention period, due to the lack of MRI data from the pre-intervention period. Taken together with available evidence showing age-related volume reductions in some regions, including the lateral prefrontal cortex and medial temporal lobe [18, 20, 69–71], the latter might be more possible. To address this issue, future research will need to acquire longitudinal data through the intervention period and make comparisons with the data from both groups. The best way to examine this possibility would be performing a two-way mixed analysis of variance (ANOVA) with a between-subjects factor of group (intervention, control) and a within-subjects factor of time (pre-intervention, post-intervention) and investigating whether the regional brain volume would show an interaction between the two factors. Despite the limitation, this pilot study achieved its purpose by identifying candidate brain regions that are responsible for the beneficial effect of conversation-based interventions on brain structures.

The other limitation is that some of the reported regions did not meet the significance criteria when additional nuisance covariates were included in the two-sample *t*-test. Specifically, when the scores of the Japanese version of the 15-item Geriatric Depression Scale (GDS-15-J) [72] after the intervention were employed as an additional nuisance covariate to adjust participants’ mental status, the lateral prefrontal cortex and medial temporal lobe did not achieve statistical significance, whereas the other regions, including the posterior middle temporal gyrus and the somatosensory cortex, remained significant. When the scores of the Japanese version of the Montreal Cognitive Assessment (MoCA-J) [26] were used to adjust participants’ global cognition, the posterior middle temporal gyrus was significant, while the other regions were not. However, when the MMSE-J scores [42] were employed, all of the reported regions met the significance criteria. Thus, note that a part of the present findings could be limited by some factors, such as mental status and/or global cognition.

The current findings imply structural changes in the brain induced by balanced conversations, in which both inputs and outputs of information are required. Practicing conversations in such a balanced manner in daily life could be an effective approach to maintain or improve cognitive and brain health, and the effectiveness may improve by introducing the proposed robotic system. Our findings support existing policies to increase opportunities for community-dwelling older adults to interact with others. However, an alternative method which has similar effects on cognitive and brain health would have special needs for practice, since some people are reluctant to meet others. Toward addressing this issue, a dialogue-based robot system for daily cognitive training without meeting other people at home has been recently developed [73]. The user of the dialogue-based robot system listens carefully to the robotic speech and asks questions based on the speech, which requires both inputs and outputs of information. As the next step, the effects of this dialogue system on cognitive health in older adults would be examined in future research.

## Conclusions

In this pilot study, we examined the difference in brain structures between the intervention and control groups after the intervention. Our results demonstrated that the volume of the superior frontal gyrus was greater in the intervention group than in the control group. Greater volume in the intervention group was also identified in the parahippocampal gyrus/hippocampus, posterior middle temporal gyrus, and postcentral gyrus. In contrast, no regions showed greater volume in the control group than in the intervention group. Furthermore, the region whose volume was positively correlated with the increase in verbal fluency scores was found in the inferior parietal lobule. This pilot study successfully identified candidate brain regions that reflect the beneficial effect of conversation-based interventions on cognitive function, including the lateral prefrontal cortex, which plays a pivotal role in executive functions. Further investigation will be needed to confirm this possible effect by collecting and comparing MRI data from the pre- and post-intervention periods in future studies.

## Supplementary Information

**Additional file 1. **Behavioral results of pre/post cognitive and mental health tests in the intervention (*n* = 31) and control (*n* = 30) groups.

**Additional file 2. **VBM outputs in the intervention (*n* = 31) and control (*n* = 30) groups.

## Data Availability

The datasets generated and/or analyzed in the present study are not publicly available due to the requirement of a joint research agreement for data sharing, but are available from the corresponding author on reasonable request.

## Abbreviations

MRI: Magnetic resonance imaging
PICMOR: Photo-Integrated Conversation Moderated by Robots
RCT: Randomized controlled trial
VBM: Voxel-based morphometry
SD: Standard deviation
MMSE-J: Japanese version of the Mini-Mental State Examination
SPM: Statistical Parametric Mapping
GM: Gray matter
WM: White matter
CSF: Cerebrospinal fluid
DARTEL: Diffeomorphic Anatomical Registration using Exponentiated Lie algebra
MNI: Montreal Neurological Institute
WMS-R: Wechsler Memory Scale-Revised
ANOVA: Analysis of variance
GDS-15-J: Japanese version of the 15-item Geriatric Depression Scale
MoCA-J: Japanese version of the Montreal Cognitive Assessment
