# Supplementary material for: A pilot voxel-based morphometry study of older adults after the PICMOR intervention program
Source: BMC Geriatr. 2022 Jan 19;22:63. doi: 10.1186/s12877-021-02669-x (PMC8772081; doi:10.1186/s12877-021-02669-x)
Supplement: Supplementary file 1 — Additional file 1. Behavioral results of pre/post cognitive and mental health tests in the intervention (n = 31) and control (n = 30) groups. [file 12877_2021_2669_MOESM1_ESM.docx]

**Supplementary Information**

**A pilot voxel-based morphometry study of older adults after the PICMOR intervention program**

Hikaru Sugimoto, Mihoko Otake-Matsuura

| Behavioral results of pre/post cognitive and mental health tests in the intervention (n = 31) and control (n = 30) groups | | | | | | | | |  |
| --- | --- | --- | --- | --- | --- | --- | --- | --- | --- |
|  | Pre-intervention | | | | Post-intervention | | | | |
|  | Intervention  (mean ± SD) | Control  (mean ± SD) | Welch’s *t*-test  (*t*, *p*, *Cohen’s d*) | Wilcoxon rank sum test  (*W*, *p*, *Cliff’s delta*) | Intervention  (mean ± SD) | Control  (mean ± SD) | Welch’s *t*-test  (*t*, *p*, *Cohen’s d*) | Wilcoxon rank sum test  (*W*, *p*, *Cliff’s delta*) | |
| MMSE-J^1)^ | 27.97 ± 1.45 | 28.10 ± 1.49 | 0.35, 0.73, 0.09 | 493.5, 0.68, 0.06 | 28.65 ± 1.80 | 28.73 ± 1.26 | 0.22, 0.82, 0.06 | 452.5, 0.86, 0.03 | |
| MoCA-J^2)^ | 25.94 ± 2.50 | 25.40 ± 2.79 | 0.79, 0.43, 0.20 | 423, 0.55, 0.09 | 26.26 ± 2.71 | 25.07 ± 2.94 | 1.65, 0.11, 0.42 | 342, 0.07, 0.26 | |
| Logical memory I (immediate)^3)^ | 10.13 ± 3.22 | 8.50 ± 4.04 | 1.74, 0.09, 0.45 | 321, 0.04, 0.31 | 11.48 ± 3.46 | 10.83 ± 4.30 | 0.65, 0.52, 0.17 | 441, 0.73, 0.05 | |
| Logical memory II (delayed) | 8.58 ± 3.03 | 6.83 ± 3.65 | 2.03, 0.05, 0.52 | 328, 0.05, 0.29 | 9.68 ± 3.41 | 9.23 ± 4.42 | 0.44, 0.66, 0.11 | 447.5, 0.80, 0.04 | |
| Verbal fluency^3)^ | 12.03 ± 3.61 | 11.13 ± 4.07 | 0.91, 0.37, 0.23 | 426.5, 0.58, 0.08 | 13.71 ± 3.55 | 11.07 ± 2.95 | 3.17, 0.00, 0.81 | 266, 0.00, 0.43 | |
| Symbol^4)^ | 55.70 ± 13.05 | 51.50 ± 14.16 | 1.19, 0.24, 0.31 | 373.5, 0.26, 0.17 | 67.23 ± 11.36 | 61.13 ± 11.00 | 2.11, 0.04, 0.55 | 346, 0.13, 0.23 | |
| Digit span forward^1)^ | 9.77 ± 2.06 | 9.70 ± 1.80 | 0.15, 0.88, 0.04 | 473.5, 0.90, 0.02 | 9.71 ± 1.66 | 10.17 ± 1.62 | 1.09, 0.28, 0.28 | 557, 0.18, 0.20 | |
| Digit span backward^3)^ | 6.45 ± 1.77 | 6.07 ± 1.39 | 0.95, 0.35, 0.24 | 382, 0.22, 0.18 | 6.48 ± 1.88 | 6.53 ± 1.55 | 0.11, 0.91, 0.03 | 482, 0.81, 0.04 | |
| TMT-A^1,5)^ | 4.21 ± 0.35 | 4.34 ± 0.54 | 1.11, 0.27, 0.29 | 480, 0.66, 0.07 | 4.12 ± 0.28 | 4.32 ± 0.51 | 1.90, 0.06, 0.49 | 525, 0.27, 0.17 | |
| TMT-B^1,5)^ | 4.74 ± 0.46 | 4.81 ± 0.56 | 0.54, 0.59, 0.14 | 471, 0.76, 0.05 | 4.54 ± 0.31 | 4.68 ± 0.44 | 1.43, 0.16, 0.37 | 518, 0.32, 0.15 | |
| GDS-15-J^1)^ | 2.03 ± 1.72 | 3.23 ± 2.16 | 2.40, 0.02, 0.62 | 316, 0.03, 0.32 | 1.97 ± 1.99 | 3.07 ± 2.20 | 2.05, 0.05, 0.52 | 319.5, 0.03, 0.31 | |

^1)^ Results of Shapiro-Wilk tests showed that assumption of normality of the intervention and/or control group was violated in both the pre- and post-intervention periods. ^2)^ Results of Shapiro-Wilk tests showed that assumption of normality of the intervention group was violated in the post-intervention period. ^3)^ Results of Shapiro-Wilk tests showed that assumption of normality of the intervention or control group was violated in the pre-intervention period. ^4)^ One participant with a missing value in the intervention group was eliminated. ^5)^ One participant with a missing value in the intervention group was eliminated. The logarithmically transformed response variable was used for analysis.

*Abbreviations: MMSE-J* Japanese version of the Mini-Mental State Examination, *MoCA-J* Japanese version of the Montreal Cognitive Assessment, *TMT* Trail Making Test, *GDS-15-J* Japanese version of the 15-item Geriatric Depression Scale, *SD* standard deviation
