# Supplementary material for: A pilot voxel-based morphometry study of older adults after the PICMOR intervention program
Source: BMC Geriatr. 2022 Jan 19;22:63. doi: 10.1186/s12877-021-02669-x (PMC8772081; doi:10.1186/s12877-021-02669-x)
Supplement: Supplementary file 2 — Additional file 2. VBM outputs in the intervention (n = 31) and control (n = 30) groups. [file 12877_2021_2669_MOESM2_ESM.docx]

**Supplementary Information**

**A pilot voxel-based morphometry study of older adults after the PICMOR intervention program**

Hikaru Sugimoto, Mihoko Otake-Matsuura

| VBM outputs in the intervention (n = 31) and control (n = 30) groups | | |
| --- | --- | --- |
|  | Intervention  (mean ± SD) | Control  (mean ± SD) |
| Gray matter volume (ℓ) | 0.5837 ± 0.0494 | 0.5577 ± 0.0514 |
| White matter volume (ℓ) | 0.4147 ± 0.0534 | 0.3949 ± 0.0444 |
| Cerebrospinal fluid volume (ℓ) | 0.4704 ± 0.0740 | 0.4623 ± 0.0940 |
| Total intracranial volume (ℓ) | 1.4685 ± 0.1457 | 1.4145 ± 0.1311 |

A two-way mixed ANOVA for the VBM outputs showed no significant main effect of group (intervention, control) (*F* [1, 59] = 2.30, *p* = 0.13) and interaction between group (intervention, control) and tissue (gray matter, white matter, cerebrospinal fluid) (*F* [2, 118] = 0.44, *p* = 0.65).

*Abbreviations: VBM* voxel-based morphometry, *SD* standard deviation, *ANOVA* analysis of variance
